# Clinicopathological study and molecular subtyping of muscle-invasive bladder cancer (MIBC) using dual immunohistochemical (IHC) markers

**DOI:** 10.1186/s13000-025-01603-8

**Published:** 2025-01-24

**Authors:** R. Vaithegi, Kanthilatha Pai, Anuradha Calicut Kini Rao, Vidya Monappa, Swathi Prabhu, Nischitha Suvarna

**Affiliations:** 1 Department of Pathology, Kasturba Medical College, India Manipal, 576104,; 2 Kasturba Medical College, Manipal, Manipal Academy of Higher Education, Manipal, 576104 Karnataka India

**Keywords:** Muscle-invasive bladder carcinoma, Molecular subtypes, Immunohistochemistry, GATA3, CK5/6, Luminal, Basal

## Abstract

**Background:**

Muscle-invasive bladder carcinomas (MIBCs) exhibit significant heterogeneity, with diverse histopathological features associated with varied prognosis and therapeutic response. Although genomic profiling studies have identified several molecular subtypes of MIBC, two basic molecular subtypes are identified - luminal and basal, differing in biological behaviour and response to treatment. As molecular subtyping is complex, surrogate immunohistochemical (IHC) markers have been used to determine the molecular subtypes with good correlation to genomic profiling.

**Methods:**

We analysed the clinicopathological features of 66 cases of MIBCs received over a 5-year study period. IHC expression was determined using GATA3 and CK5/6 to classify MIBC into luminal, basal and double-negative subtypes. The association between clinicopathologic variables and molecular subtypes were analysed using Chi-square test.

**Results:**

The mean age at diagnosis of MIBC was 65.91 years with a male predominance. Based on IHC expression of GATA3 and CK5/6, MIBCs were classified into luminal, basal and double negative subtypes in 62.1%, 30.3% and 7.6% respectively. The luminal subtype occurred at an older age and showed predominantly conventional urothelial carcinoma with papillary morphology. Basal subtype occurred at earlier age, showed greater association with smoking and was more commonly associated with urothelial carcinoma with non -papillary morphology and exhibiting divergent differentiation as well as pure squamous cell carcinoma on histopathological examination. The double-negative subtype was found exclusively in males and exhibited a non-papillary morphology. Notably, all diagnosed neuroendocrine carcinomas were classified as double-negative type. While there was no statistically significant difference in tumour stage in cystectomy specimens between the molecular subtypes, lympho-vascular invasion and lymph node metastasis was more commonly associated with the basal type (*p* < 0.05) There was no significant difference in recurrence rates, metastasis and death between luminal and basal subtypes.

**Conclusion:**

A simple two-antibody panel using GATA3 and CK5/6 could help in classifying MIBC into basic molecular subtypes of MIBC with distinctive histopathological features that can provide insights into the corresponding molecular subtype. Greater association of lymphovascular invasion and lymph nodal involvement in cystectomy specimens in basal type and distant metastasis in the double-negative subtype suggests a more aggressive clinical behaviour of these, necessitating more intensive treatment.

## Introduction

Carcinoma of urinary bladder presents with wide range of clinical features and consists of diverse histological types that influences the disease progression, prognosis, and recurrence [1]. It is a common malignancy occurring in the urinary tract, that accounted for 613,791 new cases and 220,349 cancer-related deaths in the world in 2022. In the same year, India reported 22,548 cases, constituting 10.5% of the total cases of bladder cancers in Asia, which occurred three to four times more commonly in males than females [2]. Histologically, bladder carcinomas are of several types that include urothelial carcinomas, squamous cell carcinoma, adenocarcinoma, urachal carcinoma, diverticular carcinoma, tumours of Mullerian type etc [1]. The presence or absence of detrusor muscle invasion is an important determinant of prognosis; hence they are classified into non-muscle invasive bladder cancer (NMIBC), which account for nearly 80% of cases and muscle-invasive bladder cancer (MIBC) making up the remaining cases [3]. Genomic profiling and mutational studies in NMIBCs and MIBCs have identified several molecular subtypes; the primary molecular subtypes of MIBC being luminal and basal subtypes which express markers of urothelial and basal/squamous differentiation respectively [4]. These groups are reported to be associated with distinct clinicopathological features, with basal subtype exhibiting worse prognosis with better response to chemotherapy, compared to the luminal subtype. A comprehensive analysis of these processes helps in accurate diagnosis and multimodality treatment approaches. Studies have demonstrated the utility of surrogate immunohistochemistry (IHC) markers to identify the molecular subtypes using GATA3, CK20 as luminal markers while CK5/6, CK14 as basal markers. A meta-analysis on three cohorts of bladder carcinoma (937 samples) to analyse the gene expression profiles using a panel of immunohistochemical markers, demonstrated that the two subtypes can be identified using only two markers - GATA3 and CK5/6 for luminal and basal tumours respectively, with an accuracy of more than 90% [5].

This study aimed study the clinicopathological features of MIBCs and classify them into molecular subtypes using a limited panel of two antibodies-GATA3 and CK5/6 and analyse their correlation with the biological behaviour.

## Materials and methods

### Case selection

A 5-year retrospective study (January 2017 to December 2021) of all diagnosed cases of carcinoma of the bladder with muscle invasion (MIBC) retrieved from the database of Department of Pathology was conducted, that included transurethral resection specimens of bladder tumour (TURBT) as well as radical cystectomy specimens. NMIBCs, mesenchymal tumours and lymphomas were excluded from the study. The study was approved by the institutional ethics committee (IEC2: 449/2022). The clinico-demographic data, cystoscopy findings and treatment details were obtained from the electronic medical records. Follow up details including the duration of follow-up, patient survival and recurrence data were obtained wherever available.

Hematoxylin and Eosin (H & E) slides were reviewed for histomorphological features and classified according to the World Health Organization (WHO) 5th edition and pathological staging was done as per TNM and American Joint Committee on Cancer (AJCC) guidelines.

### Immunohistochemistry

IHC was performed on all the cases using GATA3 (clone L50-823, mouse monoclonal antibody, Biocare Medical) and CK5/6 (clone CK5/6.007, mouse monoclonal antibody, Biocare Medical). The representative areas of muscle-invasive tumour were identified, marked on the slide and in the formalin-fixed paraffin-embedded blocks. Tissue microarray (TMA) was constructed by retrieving 5 mm tumour tissue cores, and each recipient block was embedded with seven cores. Manual IHC staining was performed on the tumour samples along with appropriate positive controls for both antibodies (breast cancer and prostate tissue for GATA3 and CK5/6 respectively), starting with heat-induced epitope retrieval. Primary antibody incubation was done for one hour followed by horse-radish peroxidase labelled MACH1 antibody with Betazoid diaminobenzidine as chromogen. Counterstain with hematoxylin was performed and the results were interpreted. The IHC slides were interpreted as positive if the tumour cells showed nuclear stain for GATA3 and cytoplasmic or membrane stain for CK5/6. Based on the staining percentage in the tumour cell population, they were declared positive if staining was more than 10% and negative if less than 10% similar to study by Bejrananda et al. [6]

### IHC-based molecular subtypes

Molecular subclassification using surrogate IHC markers – GATA3 and CK5/6 were done as follows.

- GATA3 positive, CK5/6 negative - Luminal subtype.
- GATA3 negative, CK5/6 positive - Basal subtype.
- GATA3 negative, CK5/6 negative - Double negative subtype.

Tumours staining for both GATA3 and CK5/6 staining, with CK5/6 observed in < 10% tumour population were grouped under the luminal subtype as some luminal tumours are shown to possess scattered CK5/6 positive cells [4].

### Statistics

Data was analysed using IBM-SPSS Statistics for Windows version 23.0 (Armonk, NY: IBM Corp). The categorical data was expressed in terms of proportions and percentages. Continuous variables were expressed using mean and standard deviation. T test was used to compare means, and Chi-square test was employed to study the association between the categorical variables. A *p* value of < 0.05 was considered statistically significant.

## Results

During the 5-year study period, 400 cases of bladder carcinomas were received, of which 66 (16.5%) cases were MIBC with detrusor muscle invasion.

Majority were TURBT samples constituting 48 cases (72.7%) while 18 cases (27.3%) were radical cystectomy specimens. The clinicopathologic features of MIBCs are summarized in Table 1.

The age of the patients ranged from 40 to 85 years with mean age at diagnosis being 65.91 years. Male to female ratio was 3.71:1. Most common clinical manifestation noted was painless haematuria in 86.4% of patients, followed by burning micturition in 40.9% patients. Cystoscopically, sites involved were lateral walls (45.5%), followed by dome (22.1%), neck of bladder (21.7%), anterior wall (19.7%), posterior wall (19.7%), trigone (16.7%), and ureteric orifice (3.0%). The lesions were solitary in most patients (75.8%) with a mean size of 2.98 cm.

Histopathological examination showed that most of the cases were conventional urothelial carcinomas in 54.5% cases, and urothelial carcinoma with divergent differentiation in 36.4% of which squamous differentiation was the most common. Non-urothelial carcinomas -squamous cell carcinoma in 6.1% and neuroendocrine carcinoma in 3% cases noted. Pathological staging and lymph node involvement were available for 18 and 11 cystectomy specimens respectively, listed in Table 1.

**Table 1:** Clinicodemographic and histopathological characteristics of the 66 MIBCs

Parameters Clinical features		*n* (%)
Age	Mean + SD	65.91 ± 9.70 years
Gender	Male	52 (78.8)
	Female	14 (21.2)
Risk factors Smoking (*n* = 27)	Yes	17 (62.96)
	No	10 (37.04)
**Cystoscopy findings**		
Number of lesions	Single	50 (75.8)
	Multiple	16 (24.2)
Size of lesion	Mean + SD	2.98 ± 2.68 cm
	Median	3 cm
	Range	01 to 10 cm
**Microscopic findings**		
Histological type	Conventional urothelial carcinoma	36 (54.5)
	Urothelial carcinoma with squamous differentiation	21 (31.9)
	Sarcomatoid type of urothelial carcinoma	2 (3.0)
	Giant cell type of urothelial carcinoma	1 (1.5)
	Neuroendocrine carcinoma	2 (3.0)
	Squamous cell carcinoma	4 (6.1)
Architecture	Papillary	30 (45.5)
	Non- papillary	36 (54.5)
Necrosis	Present	56 (84.8)
	Absent	10 (15.2)
Perineural invasion	Present	31(47.0)
	Absent	35(53.0)
Lymphovascular invasion	Present	40 (60.6)
	Absent	26 (39.4)
Stromal inflammatory infiltrate	Mild	14 (21.2)
	Moderate	41 (62.1)
	Brisk	11 (16.7)
Pathological stage (*n* = 18)	pT2	5 (27.8)
	pT3	4 (22.2)
	pT4	9 (50)
Lymph node metastasis (*n* = 11)	N0	4 (36.4)
	N1	3 (27.2)
	N2	4 (36.4)

**Fig. 1:**
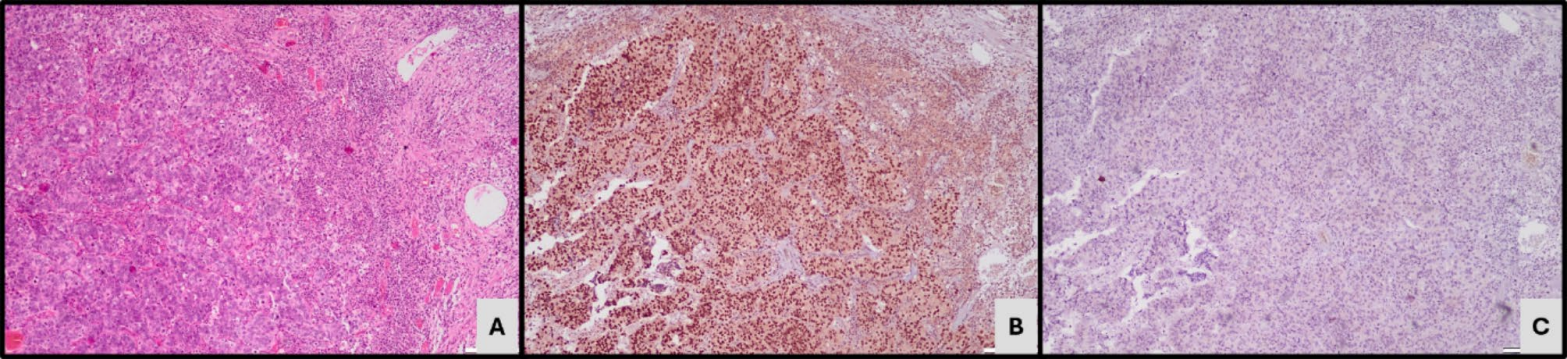
Microscopy - IHC-Luminal subtype. (**A**) Conventional urothelial carcinoma (H&E, X200). (**B**) GATA3 IHC nuclear staining (X200). (**C**) CK5/6 IHC negativity (X200)

**Fig. 2:**
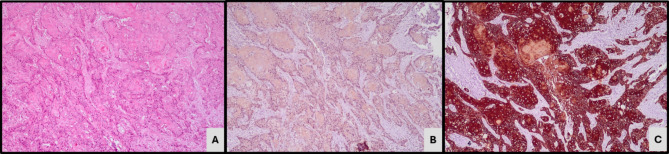
Microscopy - IHC-Basal subtype. (**A**) Squamous cell carcinoma (H&E, X200). (**B**) GATA3 IHC negativity (X200). (**C**) CK5/6 IHC cytoplasmic staining (X200)

**Fig. 3:**
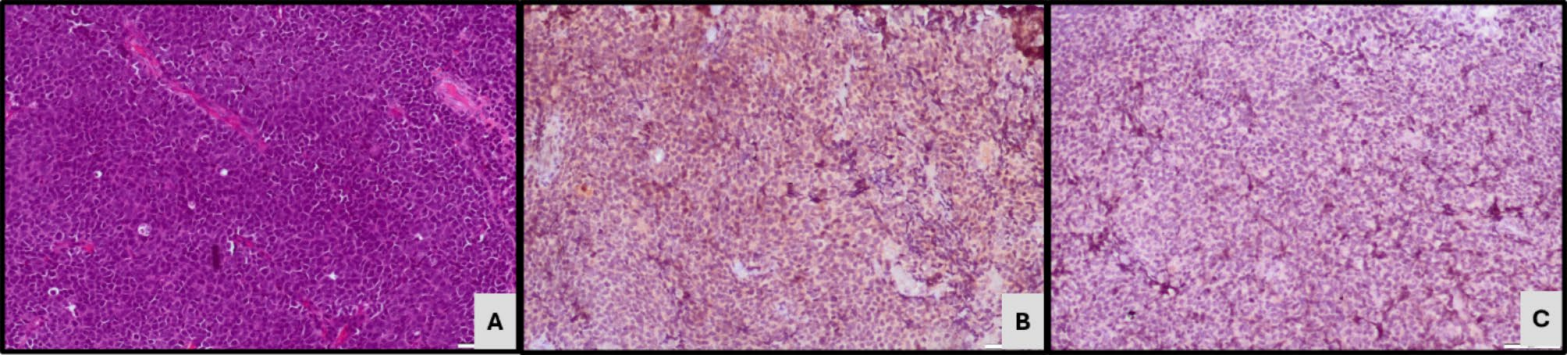
Microscopy - IHC-Double negative subtype. (**A**) Neuroendocrine carcinoma (H&E, X200). (**B**) GATA3 IHC negativity (X200). (**C**) CK5/6 IHC negativity (X200)

**Fig. 4:**
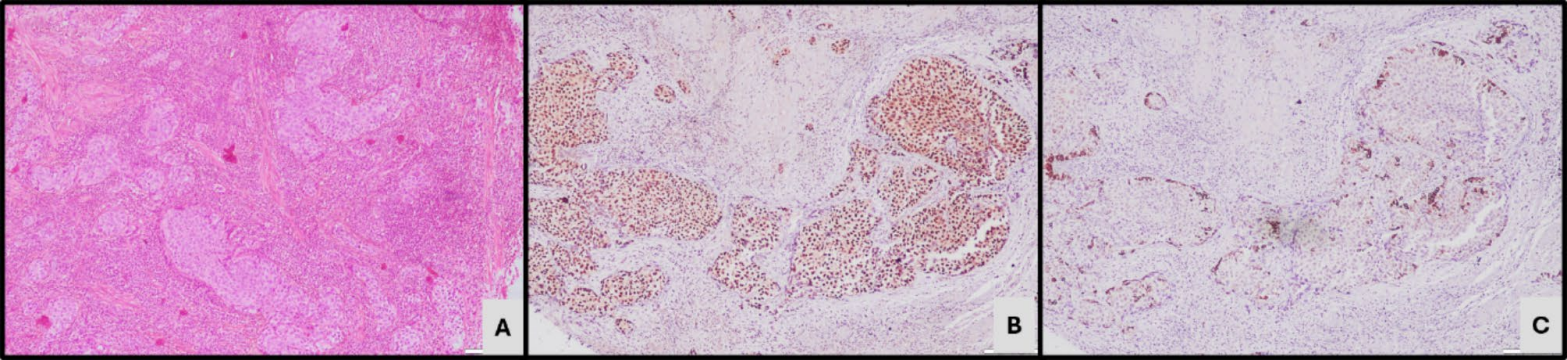
Microscopy -IHC Luminal subtype. (**A**) Conventional Urothelial carcinoma (H&E, X200). (**B**) GATA3 IHC positivity (X200). (**C**) CK5/6 IHC positivity in < 10% tumour cells (X200)

Based on the expression of GATA 3 and CK5/6 expression, 41 cases (62.1%) were classified as luminal subtype expressing GATA3 IHC (Fig. 1). 20 cases (30.3%) demonstrating CK5/6 expression as basal subtype (Fig. 2) while the remaining five (7.6%) cases did not express either of the 2 markers and were classified as the double-negative subtype (Fig. 3). Twenty-one cases which demonstrated positive GATA3 expression with scattered or basal strips of CK5/6 staining in < 10% tumour population were included in the luminal subtype (Fig. 4).

The association of the clinicopathologic features with these subtypes are given in Table 2. IHC-basal subtype presented at a younger mean age (*p* = 0.049). A male preponderance was noted among all subtypes. On cystoscopy, most cases showing multiple lesions belonged to the luminal subtype while larger lesions were associated with basal subtype. On microscopy, predominant papillary architecture was observed commonly in the luminal subtype (76.7%).

**Table 2:** Association of clinicopathological characteristics and IHC-based molecular subtypes of the 66 MIBCs

Parameters	Luminal subtype *n* = 41 (62.1)	Basal subtype *n* = 20 (30.3)	Double negative subtype *n* = 5 (7.6)	*p* value
**Age**	67.44+/- 9.98	62.70+/-9.14	66.20+/-7.82	0.049*
**Gender**				
Male (*n* = 52)	34 (65.4)	13 (25.0)	5 (9.6)	0.133
Female ((*n* = 14)	7 (50.0)	7 (50.0)	0	
**Smoking (***n* = **27)**				
Yes (*n* = 17)	11 (64.7)	6 (35.3)	0	0.433
No (*n* = 10)	5 (50.0)	3 (30.0)	2 (20.0)	
**Cystoscopy**				
**Number of lesions**				
Single (*n* = 50)	29 (58.0)	16 (32.0)	5 (10.0)	0.307
Multiple (*n* = 16)	12 (75.0)	4 (25.0)	0	
**Size of lesion**	2.66 +/- 2.46	3.70 +/- 3.08	2.80 +/- 2.77	0.363
**MICROSCOPY – Architecture**				
Papillary (*n* = 30)	23 (76.7)	6 (20.0)	1 (3.3)	0.077
Non-papillary (*n* = 36)	18 (50.0)	14 (38.9)	4 (11.1)	
**Histology**				
Conventional Urothelial Carcinoma (*n* = 36)	32(88.9)	1 (2.8)	3 (8.3)	< 0.005*
Urothelial carcinoma with squamous differentiation (*n* = 21)	8(38.0)	13 (62.0)	0	
Urothelial Carcinoma – Sarcomatoid type (*n* = 2)	1(50.0)	1 (50.0)	0	
Urothelial Carcinoma- Giant cell type (*n* = 1)	0	1 (100.0)	0	
Squamous Cell Carcinoma (*n* = 4)	0	4 (100.0)	0	
Neuroendocrine carcinoma (*n* = 2)	0	0	2 (100.0)	
**Necrosis**				
Present (*n* = 56)	34 (60.7)	17 (30.4)	5 (8.9)	0.603
Absent (*n* = 10)	7 (70.0)	3 (30.0)	0	
**Lymphovascular invasion**				
Present (*n* = 23)	7 (30.4)	13 (56.6)	3 (13.0)	< 0.05*
Absent (*n* = 43)	34 (79.0)	7 (16.3)	2 (4.7)	
**Perineural invasion**				
Present (*n* = 31)	16 (51.6)	14 (45.2)	1 (3.2)	0.034*
Absent (*n* = 35)	25 (71.4)	6 (17.1)	4 (11.5)	
**Stromal inflammatory infiltrate**				
Mild (*n* = 14)	9 (64.2)	3 (21.4)	2 (14.4)	0.222
Moderate (*n* = 40)	26 (65.0)	11 (27.5)	3 (7.5)	
Brisk (*n* = 12)	6 (50.0)	6 (50.0)	0	
**Pathological staging (***n* = **18)**				
T2 (*n* = 5)	3 (60.0)	2(40.0)	0	0.609
T3 (*n* = 4)	2 (50.0)	2 (50.0)	0	
T4 (*n* = 9)	3 (33.3)	6 (66.7)	0	
**Lymphnode involvement (***n* = **11)**				
N0 (*n* = 4)	4 (100.0)	0	0	0.022*
N1 /N2 (*n* = 7)	2 (28.6)	5 (71.4)	0	
**Follow up data (***n* = **17)**				
Recurrence (*n* = 2)	1 (50.0)	1 (50.0)	0	0.065
Metastasis (*n* = 6)	3 (50.0)	0	3 (50.0)	
Death (*n* = 9)	4 (44.5)	5 (55.5)	0	

*p value less than 0.05 considered statistically significant

Conventional urothelial carcinomas was identified to be predominantly of luminal subtype (88.9%) while urothelial carcinomas with squamous differentiation and subtypes and pure squamous cell carcinomas (SCC) belonged to the basal subtype on histopathological examination.

Although pathological staging in 18 cases of MIBC in cystectomy specimens revealed most pT4 cases to be of basal subtype (66.7%), there was no statistically significant difference in pathological staging between the IHC molecular subtypes. Necrosis was a common feature seen in all the 3 subtypes, with all double-negative cases demonstrating it. Perineural invasion (PNI) was noted in both luminal and basal subtypes, with slight preponderance in luminal cases. In contrast, lymphovascular invasion (LVI) (*p* < 0.05) and lymph node involvement (*p* < 0.022) were noted more commonly with basal subtype.

Patients were followed up for a period of three years which was available only for 17 patients, rest were lost to follow up. Two cases (one luminal, one basal type) showed recurrence. Luminal subtype of the high grade conventional urothelial carcinoma showed similar features on recurrence as the primary tumor, while the basal type of high-grade urothelial carcinoma showed divergent sarcomatoid differentiation in the recurrent tumor. Distant metastasis to bone, lung and liver was seen in three patients of luminal subtype and double negative subtype respectively. Post-radical cystectomy, eight patients died due to post-operative complications such as aspiration pneumonia, acute pulmonary embolism and sepsis.

## Discussion

Bladder cancers are heterogeneous at the molecular level with distinct pathways involved in pathogenesis of NMIBCs and MIBCs. Various groups have studied the genomic landscape across bladder tumours, utilizing individual study cohorts that helped to stratify the patients for prognosis and to predict response to neoadjuvant chemotherapy. Several classifications have categorised both NMIBCs and MIBCs into various molecular subtypes using mRNA assays or IHC markers. Each system identified different clusters, starting with two subtypes (luminal-like and basal-like) and recently leading to the development of the consensus classification system identifying six molecular subtypes: luminal-papillary, luminal unstable, luminal non-specified, stroma-rich, basal/squamous neuroendocrine-like [7, 8]. Studies have indicated that the gene expression profiles of the two primary molecular subtypes: luminal and basal subtypes mirror the expression patterns of normal luminal and basal urothelial cells. Using surrogate IHC markers, bladder tumours can be classified as luminal when they express GATA3, and CK20, and basal when they show expression of CK5/6 and CK14 [5]. To effectively incorporate the classification system into clinical practice, surrogate IHC analysis plays an important role, especially in resource-poor settings.

MIBCs presented commonly in the elderly population in our study with mean age of 65.91 years, comparable to the findings of other studies [6, 9, 10]. Most participants were males, and a similar male preponderance was noted by various authors [3, 6, 10, 11]. Data on smoking history revealed 63% (17/27) to be smokers. Other studies also reported smoking in 71.4% and 71% participants respectively, thus indicating a strong relationship between smoking and bladder carcinoma [12, 13]. Cystoscopy noted most lesions to be solitary (75.8%) with a mean size of 2.98 cm, like the findings of Satoh E et al. where 72.2% of MIBCs were found to be solitary measuring ≥ 1 cm [14]. The commonest sites involved were the lateral walls followed by the dome, trigone similar to the study by Stephenson et al. who noted 37.1% occurring in lateral wall followed by posterior walls and trigone [15].

Histopathological examination showed papillary architecture in 45.5% of all tumours, in contrast to only 25% noted by Jangir H et al. [3]. Most tumours were conventional urothelial carcinomas followed by urothelial carcinomas with squamous differentiation, as also noted in various studies [3, 10, 11, 16] while SCC was seen in higher numbers in study by Naga NK et al. [17]. Only small numbers of giant cell and sarcomatoid subtypes as well as neuroendocrine carcinomas were identified in our study, coinciding with other studies [11, 17, 18].

Our study demonstrates that GATA3 and CK5/6 immunostaining was noted in 41 and 20 cases respectively.

The luminal subtype constituted 62.1% of the study population. A meta-analysis on transcriptome expression identified 70% and 81.5% luminal tumours in the MD Anderson and Lund cohort, comprising of both NMIBCs and MIBCs while a lower 52% was noted in The Cancer Genome Atlas (TCGA) cohort including only MIBCs [5]. Olkhov Mitsel et al. reported a higher (78.8%) number under the IHC-based luminal subtype which could be because the cases expressing both GATA3 and CK5/6 were included in this molecular subtype [11] while lower numbers were seen in various other studies which identified mixed subtype as a separate group [3, 6, 17].

The basal subtype was demonstrated in 30.3% MIBCs in our study which was comparable to the 26% of the MD Anderson cohort, while TCGA and the Lund cohort reported higher (44%) and lower (13.3%) basal tumours respectively [5]. Other studies have also demonstrated similar results in MIBCs [10, 11].

The double negative subtype was observed in only 7.6% of our study population, coinciding with the observations in the MD Anderson, Lund and TCGA cohort (4%, 5% and 4% respectively) [5].

The luminal subtype was associated with the following characteristics: presentation at an older age compared to other subtypes while other studies identified this group to present earlier [6, 11]. Of the 17 with a history of smoking, 11 were identified to be smokers coinciding with the findings of Sun X et al. [19]. On microscopy, papillary morphology was appreciated predominantly in this subtype (76.7%), which was comparable to the 72.7% tumours of the luminal papillary, luminal non-specified and luminal unstable subtypes studied by Benitez et al. [20]. Most cases of conventional urothelial carcinoma along with few showing divergent squamous differentiation were associated with luminal subtype, as was seen in numerous analyses [10, 11, 18].

The basal subtype significantly occurred at an earlier age (mean − 62.70) than other subtypes, like other studies [3, 10]. The female patients in the study population were equally distributed between basal and luminal subtypes, in contrast to the female predominance demonstrated in other studies [3, 10]. Smoking was observed in 6/9 basal MIBCs, which is consistent with research demonstrating significant association between smoking and basal subtype [19]. Histopathological examination revealed the basal subtype associated with non-papillary morphology as was demonstrated by other studies [20, 21]. Urothelial carcinoma with squamous differentiation and pure squamous cell carcinoma were of this basal subtype which was statistically significant. Similar findings were also observed by other researchers [10, 11]. All the cases of urothelial carcinoma - giant cell type, squamous cell carcinoma and neuroendocrine carcinoma were negative on IHC for GATA3, which agreed with the results of Naga NK et al. [17]. LVI was noted in most cases, like other studies [6, 10] while Sanguedolce et al. showed lower LVI in the basal subtype [21]. PNI was observed in more than half the cases, reinforcing the findings of Serag Eldien MM et al. that PNI was significantly associated with cases showing CK5/6 positivity and low GATA3 expression [16]. Moderate to brisk stromal inflammatory infiltrate was identified, which was in concordance with studies showing high stromal infiltration in the basal/squamous subtype [7, 20].

In our study, the five cases of double-negative subtype were all males, aligning with other studies [6]. No smokers were noted in this group. Histology showed three cases of conventional urothelial carcinoma, predominantly non-papillary type and two neuroendocrine carcinomas. This lack of expression of both markers in neuroendocrine carcinoma was also shown in other studies [17, 18] while some studies noted GATA3 expression [22, 23]. All cases demonstrated necrosis. Brisk stromal inflammatory infiltrate was not identified in this subtype, like the findings in other studies [7].

Six cases of pT4 stage in cystectomy specimens were of the basal subtype (66.7%) similar to findings of other studies showing basal tumours mostly being pT3 and T4 cases [3, 6, 18, 21]. Lymph node involvement was also more commonly seen in basal subtype like the findings noted by other researchers [3, 10].

Limited followup data was available for only 17 patients. The two patients who developed recurrence belonged to the luminal and basal subtypes, while those with distant metastasis were seen in the double negative and luminal subtype. Bejrananda et al. noted the absence of CK5/6 or GATA3 expression to be associated with poor survival and strongly predicted a negative outcome [6]. The basal subtype showed a poor outcome with one patient showing tumour recurrence while five patients expired during the follow-up period mainly due to post-operative morbidities. The adverse outcome in the basal subtype is usually attributed to GATA3 loss, promoting tumour suppressor gene downregulation and transition from epithelial to mesenchymal type leading to increased invasiveness and spread of the tumour [24]. Helal DS and co-workers noted that most basal subtype patients in their study cohort presented with higher T stage with the presence of lymphovascular invasion and nodal involvement, contributing to the unfavorable outcome, similar to our study [10]. The worst outcome was reported in the double negative group, with a higher risk of death [6, 18].

The limitations of this study are the retrospective design in a single-centre setting and the availability of limited follow-up data due to which survival studies could not be performed. TMA was utilized to perform GATA3 and CK5/6 IHC analysis. While this method is cost and time-effective, it utilizes only a small area of the tumour tissue for staining and may not reflect the tumour heterogeneity seen commonly in bladder carcinomas. Further workup using gene profiling in a larger cohort to validate the IHC-based molecular subtypes was not done.

## Conclusion

MIBCs have been documented to have genomic instability and multiple mutations, resulting in poor prognosis in these patients, despite intensive surgery and chemotherapy. Many researchers have demonstrated the prognostic and predictive beneficial information obtained by ascertaining the molecular subtypes and immunophenotypes as regards to patient management. To effectively incorporate the classification system into clinical practice, surrogate IHC analysis plays an important role in identifying the molecular subtypes.

Accurate identification of histological variants, molecular subtypes, and immunophenotypes of urothelial carcinomas helps in risk stratification according to biological aggressiveness. This data will also provide important information about patient treatment and prognostication.

## Data Availability

The datasets analysed during the current study are available from the corresponding author on reasonable request.

## Abbreviations

AJCC: American joint committee on cancer
H&E: Hematoxylin & eosin
IHC: Immunohistochemistry
LVI: Lymphovascular invasion
MIBC: Muscle–invasive bladder carcinoma
NMIBC: Non–muscle invasive bladder carcinoma
PNI: Perineural invasion
SCC: Squamous cell carcinoma
TCGA: The cancer genome atlas
TMA: Tissue microarray
TURBT: Transurethral resection of bladder tumour
WHO: World health organization

## References

[1] WHO Classification of Tumours Editorial Board. Urinary and male genital tumours. Lyon (France): International Agency for Research on Cancer; 2022 [cited 2024 09 30]. (WHO classification of tumours series, 5th ed.; vol. 8). Available from: https://tumourclassification.iarc.who.int/chapters/36

[2] Bray F, Laversanne M, Sung H, Ferlay J, Siegel RL, Soerjomataram I, et al. Global cancer statistics 2022: GLOBOCAN estimates of incidence and mortality worldwide for 36 cancers in 185 countries. CA Cancer J Clin. 2024;74(3):229–63. 38572751 10.3322/caac.21834

[3] Jangir H, Nambirajan A, Seth A, Sahoo RK, Dinda AK, Nayak B, et al. Prognostic Stratification of Muscle Invasive Urothelial Carcinomas Using Limited Immunohistochemical Panel of Gata3 and cytokeratins 5/6, 14 and 20. Ann Diagn Pathol. 2019;43:151397–404. 31494492 10.1016/j.anndiagpath.2019.08.001

[4] Guo CC, Bondaruk J, Yao H, Wang Z, Zhang L, Lee S, et al. Assessment of Luminal and basal phenotypes in bladder Cancer. Sci Rep. 2020;10(1):9743. 32546765 10.1038/s41598-020-66747-7 PMC7298008

[5] Dadhania V, Zhang M, Zhang L, Bondaruk J, Majewski T, Siefker-Radtke A, et al. Meta-analysis of the luminal and basal subtypes of bladder Cancer and the identification of signature immunohistochemical markers for clinical use. EBioMedicine. 2016;12:105–17. 27612592 10.1016/j.ebiom.2016.08.036 PMC5078592

[6] Bejrananda T, Kanjanapradit K, Saetang J, Sangkhathat S. Impact of immunohistochemistry-based subtyping of GATA3, CK20, CK5/6, and CK14 expression on survival after radical cystectomy for muscle-invasive bladder cancer. Sci Rep. 2021;11(1):21186. 34707176 10.1038/s41598-021-00628-5 PMC8551252

[7] Kamoun A, De Reyniès A, Allory Y, Sjödahl G, Robertson AG, Seiler R, et al. A Consensus Molecular classification of muscle-invasive bladder Cancer. Eur Urol. 2020;77(4):420–33. 31563503 10.1016/j.eururo.2019.09.006 PMC7690647

[8] Zhu S, Yu W, Yang X, Wu C, Cheng F. Traditional classification and novel subtyping systems for bladder cancer. Front Oncol. 2020;10:102. 32117752 10.3389/fonc.2020.00102 PMC7025453

[9] Wang CC, Tsai YC, Jeng YM. Biological significance of GATA3, cytokeratin 20, cytokeratin 5/6 and p53 expression in muscle-invasive bladder cancer. PLoS ONE. 2019;14(8):e0221785. 31469885 10.1371/journal.pone.0221785 PMC6716637

[10] Helal DS, Darwish SA, Awad RA, Ali DA, El-Guindy DM. Immunohistochemical based molecular subtypes of muscle-invasive bladder cancer: association with HER2 and EGFR alterations, neoadjuvant chemotherapy response and survival. Diagn Pathol. 2023;18(1):11. 36737799 10.1186/s13000-023-01295-y PMC9896690

[11] Olkhov-Mitsel E, Hodgson A, Liu SK, Vesprini D, Xu B, Downes MR. Three-antibody classifier for muscle invasive urothelial carcinoma and its correlation with p53 expression. J Clin Pathol. 2021;75(11):766–71. 34103388 10.1136/jclinpath-2021-207573

[12] Bicchetti M, Simone G, Giannarini G, Girometti R, Briganti A, Brunocilla E, et al. A novel pathway to detect muscle-invasive bladder cancer based on integrated clinical features and VI-RADS score on MRI: results of a prospective multicenter study. Radiol Med. 2022;127(8):881–90. 35763251 10.1007/s11547-022-01513-5 PMC9349064

[13] Catto JWF, Rogers Z, Downing A, Mason SJ, Jubber I, Bottomley S, et al. Lifestyle factors in patients with bladder cancer: a contemporary picture of tobacco smoking, electronic cigarette use, body mass index, and levels of physical activity. Eur Urol Focus. 2023;9(6):974–82. 37080801 10.1016/j.euf.2023.04.003

[14] Satoh E, Miyao N, Tachiki H, Fujisawa Y. Prediction of muscle invasion of bladder cancer by cystoscopy. Eur Urol. 2002;41(2):178–81. 12074406 10.1016/s0302-2838(01)00035-5

[15] Stephenson WT, Holmes FF, Noble MJ, Gerald KB. Analysis of bladder carcinoma by subsite. Cystoscopic location may have prognostic value. Cancer. 1990;66(7):1630–5. 2208014 10.1002/1097-0142(19901001)66:7<1630::aid-cncr2820660730>3.0.co;2-7

[16] Serag Eldien MM, Abdou AG, Elghrabawy GRA, Alhanafy AM, Mahmoud SF. Stratification of urothelial bladder carcinoma depending on immunohistochemical expression of GATA3 and CK5/6. J Immunoass Immunochem. 2021;42(6):662–78. 10.1080/15321819.2021.1937212 34106817

[17] Naga NK, Mina SN, El-Saka AM, El-Shorbagy SH, Amer AI. Immunohistochemical expression of GATA3, CK5/6 and snail-1 in intrinsic subtypes of bladder carcinoma. Onkol Radioter. 2023;17(9):394–400.

[18] Koll FJ, Schwarz A, Köllermann J, Banek S, Kluth L, Wittler C, et al. CK5/6 and GATA3 defined phenotypes of muscle-invasive bladder Cancer: impact in Adjuvant Chemotherapy and Molecular Subtyping of negative cases. Front Med. 2022;9:875142. 10.3389/fmed.2022.875142 PMC9243590 35783619

[19] Sun X, Hoadley KA, Kim WY, Furberg H, Olshan AF, Troester MA. Age at diagnosis, obesity, smoking, and molecular subtypes in muscle-invasive bladder cancer. Cancer Causes Control. 2017;28(6):539–44. 28321693 10.1007/s10552-017-0885-z PMC5477976

[20] Benítez R, Yu K, Sirota M, Malats N, Pineda S. Characterization of the tumor-infiltrating immune repertoire in muscle invasive bladder cancer. Front Immunol. 2023;14:986598. 36817478 10.3389/fimmu.2023.986598 PMC9936234

[21] Sanguedolce F, Falagario UG, Zanelli M, Palicelli A, Zizzo M, Ascani S, et al. Clinicopathological Features and Survival Analysis in Molecular subtypes of muscle-invasive bladder Cancer. Int J Mol Sci. 2023;24(7):6610. 37047581 10.3390/ijms24076610 PMC10095107

[22] Mahmood H, Fatima H, Faheem M. Management of small cell neuroendocrine carcinoma of urinary bladder, a Case Series from Northern Pakistan. Cancer Ther Oncol Int J. 2018;9(1):13–6.

[23] Verduin L, Mentrikoski MJ, Heitz CT, Wick MR. The utility of GATA3 in the diagnosis of urothelial carcinoma with variant morphologic patterns. Appl Immunohistochem Mol Morphol. 2016;24(7):509–13. 26317312 10.1097/PAI.0000000000000221

[24] Li Y, Ishiguro H, Kawahara T, Miyamoto Y, Izumi K, Miyamoto H. GATA3 in the urinary bladder: suppression of neoplastic transformation and down-regulation by androgens. Am J Cancer Res. 2014;4(5):461–73. 25232488 PMC4163611

